# The block design subtest of the Wechsler adult intelligence scale as a possible non-verbal proxy of cognitive reserve

**DOI:** 10.3389/fnagi.2023.1099596

**Published:** 2023-03-02

**Authors:** Graciela Corujo-Bolaños, Roraima Yánez-Pérez, Nira Cedrés, Daniel Ferreira, Yaiza Molina, José Barroso

**Affiliations:** ^1^Faculty of Health Sciences, University Fernando Pessoa Canarias, Las Palmas, Spain; ^2^Faculty of Psychology, University of La Laguna, La Laguna, Spain; ^3^Division of Clinical Geriatrics, Department of Neurobiology, Care Sciences, and Society, Centre for Alzheimer Research, Karolinska Institutet, Stockholm, Sweden; ^4^Department of Psychology, Sensory Cognitive Interaction Laboratory (SCI-lab), Stockholm University, Stockholm, Sweden

**Keywords:** cognitive reserve, cognition, non-verbal abilities, WAIS-III block design subtest, WAIS-III information subtest

## Abstract

**Objectives:**

To investigate the potential of the Block design subtest of the Wechsler Adults Intelligence Scale as a non-verbal proxy of cognitive reserve.

**Method:**

A total of 391 cognitively unimpaired participants were included in this study. The association between the Block design subtest and the Information subtest (an established verbal proxy of cognitive reserve) from the WAIS, as well as the association of the two subtests with a Cognitive Reserve Questionnaire (CRQ) were tested. In addition, multiple linear regression models were conducted to investigate the association of the Block design and Information subtests with cognitive performance. The capacity of the Block design subtest to minimize the negative effect of an older age over cognitive performance was also assessed and this effect was compared with that of the Information subtest. The four cognitive domains included were: verbal memory, visual–visuospatial memory, executive-premotor functions and processing speed.

**Results:**

The Block design subtest correlated positively with both the Information subtest and the CRQ. A statistically significant association was observed between the Block design subtest and all four cognitive domains. Higher scores in the Block design subtest minimized the negative effect of aging on the cognitive domains of visual–visuospatial memory and executive-premotor functions, in a similar way to the results obtained for the Information subtest.

**Conclusion:**

The Block design subtest is significantly correlated with two established proxies of cognitive reserve: it correlates with cognitive performance and high scores in Block design have the capacity to minimize the negative effect of an older age on cognitive performance. Therefore, the results suggest that the corrected Block design subtest could be considered as a non-verbal proxy of cognitive reserve.

## 1. Introduction

Cognitive reserve (CR) refers to “the adaptability of cognitive processes that helps to explain differential susceptibility of cognitive abilities or day-to-day function to brain aging, pathology, or insult” (Stern et al., 2020). This concept helps to explain individual differences in cognitive or functional decline related to aging or brain disease (Stern et al., 2020). It has been suggested that CR is related to compensatory mechanisms (Gonzalez-Burgos et al., 2020), partially related to age differences in brain activation (Festini et al., 2018).

Research on CR has primarily focused on verbal proxies, which are presumably related to the left hemisphere of the brain (Springer et al., 2005; Bartrés-Faz et al., 2009; Solé-Padullés et al., 2009; Bosch et al., 2010; Steffener et al., 2011). Thus, research on the right hemisphere and its cognitive functions has been limited. However, the “right hemi-aging model” (RHAM) suggests that the right hemisphere is more vulnerable to aging than the left hemisphere (Dolcos et al., 2002).

Research on the right hemisphere and its functions has increased over the last few decades. In a recent systematic review (Cocquyt et al., 2017), it was suggested that the right hemisphere could have a role in compensatory processes, such as early spontaneous language recovery in aphasia. In addition, some authors have suggested the existence of a CR network within the right hemisphere (Robertson, 2014). Brosnan et al. (2018) found an association between a high CR and an increased participation of the right hemisphere in visual processing. Similarly, Fleck et al. (2017) highlighted the role of the right hemisphere in people showing better global brain functioning and high CR.

Among proxies of CR, different measures of premorbid intelligence quotient (IQ) have been widely used to operationalize CR (Stern et al., 2020). These premorbid IQ proxies typically include several subtests of the Wechsler Adult Intelligence Scale (WAIS, Wechsler, 1997), including the Information subtest (Ferreira et al., 2017; Gonzalez-Burgos et al., 2020), the Vocabulary subtest (Solé-Padullés et al., 2009), the Similarity subtest and the Digit Span subtest (Colombo et al., 2018). For instance, the Information subtest is commonly considered as a measure of crystallized intelligence (Wechsler, 2008). In the cohort used in the present study, the Information subtest showed the greatest mediation capacity for the effect of cortical thinning on cognition, as compared with the Vocabulary subtest and other proxies of CR (Ferreira et al., 2016). Furthermore, the Information subtest better represented achievements and/or use of educational opportunities versus educational attainment or years of education (Correia et al., 2015).

Hence, with regards to premorbid IQ proxies of CR, measures presumably related to the left hemisphere have also been used more. To the best of the authors’ knowledge, how non-verbal or visual (right-hemisphere) premorbid IQ proxies relate to CR has not been studied. The Block design subtest is an IQ measure of the WAIS that assesses visual domains such as visuoperceptive, visuospatial and visuoconstructive functions (Wechsler, 1997). The Block design subtest is one of the most sensitive tests to assess brain damage (Lezak et al., 2012) and it has a fluid component strongly influenced by age (Wechsler, 2008). Therefore, the Block design subtest is considered as a measure of fluid intelligence (Wechsler, 2008). As opposed to crystallized intelligence, fluid intelligence refers to the capacity for logical reasoning and the ability to solve novel problems regardless of previously acquired knowledge, typically assessed through non-verbal cognitive tests (Cattell, 1971; Yuan et al., 2018). Furthermore, the Block design subtest correlates with CR proxies such as years of education, WAIS-III Vocabulary and Information subtests and CR questionnaires (Ferreira et al., 2016).

The main objective of this study was to determine the potential role of the Block design subtest as a possible non-verbal and presumably right-hemisphere-related proxy of CR. The similarity between the Block design subtest performance and the Information subtest performance was tested, the latter being a widely used verbal IQ proxy of CR. Firstly, the Block design association with CR proxies needs to be demonstrated. In order to do this, the association between the Block design and Information subtests was tested, as well as the association of both CR proxies with an established questionnaire of CR. Secondly, the fact that the Block design and Information subtests are similarly associated with performance in different cognitive domains needs to be demonstrated. Thirdly, the capacity of Block design to minimize the negative effect of older age over cognitive performance on cross-sectional data was tested; in this case, this subtest should be considered as a CR proxy. This effect should then be compared to that of the Information subtest. The main hypothesis of the present study is that the referred Block design subtest would perform similarly to the Information subtest, demonstrating its capacity as a proxy of CR. This could have clinical implications for neuropsychological assessment and interpretation of test results, providing a non-language-dependent alternative to traditional verbal CR proxies.

## 2. Materials and methods

### 2.1. Participants

A total of 391 cognitively unimpaired participants from the GENIC database (Group of Neuropsychological Studies of the Canary Islands) were included (Ferreira et al., 2017). All participants underwent a comprehensive assessment covering global cognition, daily living activities, clinical variables, and neuropsychological tests as described in section 2.2 “Cognitive assessment.” The inclusion criteria for the current study were: (1) Age between 35 and 80 years; (2) Right-handed manual preference assessed with the Edinburgh Handedness Inventory (EHI) and additionally supported in the interview to ensure that all participants were born right-handed. This criterion was included because the authors wanted to investigate Block design as a right-hemisphere proxy of CR, and visual abilities such as those assessed by the Block design subtest are frequently driven by the right hemisphere in right-handed people, as compared with left-handed people; (3) Normal performance in comprehensive neuropsychological assessment using pertinent clinical normative data (i.e., individuals did not fulfill cognitive criteria for mild cognitive impairment or dementia); (4) Preserved global cognitive performance and normal activities of daily living based on a Mini-Mental State Examination (MMSE; Folstein et al., 1975) score ≥ 24, a Blessed Dementia Scale (BDRS; Blessed et al., 1968) score < 4 and/or a Functional Activity Questionnaire (FAQ; Pfeffer et al., 1982) score < 6; (5) No neurologic or psychiatric disorders, systemic diseases with neuropsychological consequences, history of substance abuse or use of medicines that may affect normal cognitive functioning. Following previous studies (Machado et al., 2018; Gonzalez-Burgos et al., 2020), an exception was made for the BDRS. Although the BDRS scale cut-off for abnormality is frequently established at ≥ 4 points (Blessed et al., 1968; Erkinjuntti et al., 1988), emotional factors driving the ‘changes in personality, interests and drive’ subscale may influence the BDRS total score and do not necessary reflect functional impairment. With the objective of excluding individuals with functional impairment, participants showing total BDRS scores ≥ 4 (*n* = 24) were included if: (a) 70% or higher percentage of the BDRS total score resulted from the ‘changes in personality, interests and drive’ subscale; and (b) if a score ≤ 1.5 was obtained in the other two BDRS subscales (‘changes in performance of everyday activities’ and ‘changes in habits’).

### 2.2. Cognitive assessment

The neuropsychological protocol was administered by following two alternative orders (form A and B) to avoid fatigue effects influence on specific tests. This assessment was carried out in two 3-h sessions with a 30 min break. While most tests were administered as per their standard procedures, some minor modifications were made to better fit the specific population here, as previously described (Ferreira et al., 2017). The following cognitive domains were assessed: processing speed, attention, executive functions, verbal and visual episodic memory, procedural memory, and visuoconstructive, visuoperceptive and visuospatial functions. The specific neuropsychological tests and the detailed information about the full neuropsychological assessment protocol can be found in Table 1 and in Ferreira et al. (2014).

**Table 1:** Cognitive variables and cognitive domains.

Cognitive variables		Cognitive domains
LM A-Immediate	TAVEC 1st trial	Verbal memory domain
LM B1-Immediate	TAVEC Learning	
LM B2-Immediate	TAVEC Interference	
LM A-Delay	TAVEC Short delay	
LM B-Delay	TAVEC Short delay-Clues	
LM A-Recognition	TAVEC Long delay	
LM B-Recognition	TAVEC Long delay-Clues	
VR I-Total Score	8/30 Long delay Visual	Visual memory–Visuospatial domain
VR II-Total Score	FRT	
VR-Copying	JLOT-First half	
VR-Total Recognition	JLOT-Second half	
8/30 1st trial	BNT	
8/30 Learning	Spatial Span backward	
8/30 Interference	Block Design Total	
8/30 Short delay		
STROOP Words	Digit Span	Executive functions–Premotor functions domain
STROOP Colors	Digit Span backward	
STROOP Inhibition	Spatial Span forward	
Phonemic fluency	Luria’s HAM Right	
Semantic fluency	Luria’s HAM Left	
Action fluency	Luria’s—Coordination	
PCV Decision time		Processing speed domain
PCV Motor time		
CTT-Part 1		

The table shows the cognitive variables included in each of the four different cognitive domains. Cognitive domains from a data-driven modular analyses in Garcia-Cabello et al. (2021). LM, Logical Memory; VR, Visual Reproduction; FRT, Facial Recognition Test; JLOT, Judgment of Line Orientation Test; BNT, Boston Naming Test; PCV, PC-Vienna System; CTT, Color Trails Test. HAM, Hand Motor Alternations.

Participation was voluntary and all the participants gave their written informed consent in accordance with the Declaration of Helsinki. The study was approved by the ethics committee of University of La Laguna (Spain).

The Information subtest of the WAIS-III was used as a verbal CR proxy. Participants were verbally presented with 28 questions about general knowledge (common facts, objects, places, historical figures, etc.). The Information subtest showed a high correlation with the “G” factor and with crystallized intelligence measures (Correia et al., 2015).

The Block design subtest of the WAIS-III was used as a non-verbal CR proxy. Participants were presented with 14 designs characterized by an increasing number of blocks (from 4 to 9 blocks).

The Cognitive Reserve Questionnaire (CRQ; Solé-Padullés et al., 2009; Rami et al., 2011) was additionally used as an established non-cognitive proxy of CR. The CRQ provides a global score of CR by combining information about formal educational attainment of both the participants and their parents, training courses, occupational attainment, musical training, languages, reading habits and mentally stimulating activities (e.g., chess, puzzles, crosswords, etc.). CRQ scores range from 0 to 25 in the version used in the present study (Rami et al., 2011), with higher values reflecting higher CR.

### 2.3. Statistical analysis

An important step was introduced before the main statistical analysis to adjust the Block design raw score. Age has a marked influence on the performance of the Block design subtest (Lezak et al., 2012), which reflects its fluid component (Wechsler, 2008). This poses a challenge to CR studies because the fluid component of cognition is usually the outcome variable (i.e., CR minimizes the effect of age or pathology on cognitive performance: fluid component). In contrast, the premorbid crystallized component of cognition usually remains stable with aging and in pathology (reflecting fundamental features of CR). Therefore, the age-related variability in the performance of the Block design subtest was statistically removed as a reasonable attempt to remove the fluid component of the test while retaining its crystallized component. In order to do this, the formula proposed by Amato et al. (2006) was used. This formula allows for the subtraction of the influence of one variable (age in this case) from the raw scores of the variable of interest (the Block design subtest in this case). To do this, a linear regression model including the raw score from the Block design subtest as the criterion variable and age as the predictor was performed. After obtaining the values of the parameters, the following formula was applied across all the study participants (*N* = 391):


$$\begin{array}{l} \text{Corrected score}=\text{raw}\;\text{score of the Block design subtest}-{ß}_{\text{age}}\\ \;*\left(\text{age}-\text{mean}\;\text{age}\;\text{of the cohort}\right). \end{array}$$

The new adjusted variable was used in the statistical analyses and will be hereinafter referred to as ‘the corrected Block design subtest’.

In order to address the first aim of the study, Pearson correlations were performed between the Block design subtest and the Information subtest, as well as correlations between the two subtests and the CRQ. Multiple linear regression models were conducted to address the second and third aims in this study: to investigate the association of the Block design and Information subtests with cognitive performance (second aim); and to test the capacity of Block design to minimize the negative effect of an older age over cognitive performance and compare this effect with that of the Information subtest (third aim). These models were performed separately for the Block design and Information subtests, which were included together with age as the predictors, and cognitive performance as the outcome. The main interest in the present study was the interaction term between age and the Block design/Information subtests (third aim), i.e., to investigate the capacity of the Block design and Information subtests to minimize the effect of age on cognitive performance. Although continuous variables were included in the multiple regression models, the predictor variables were dichotomized using their median values to visualize statistical interactions. In addition, to fully characterize the differences/similarities between the Block design and Information subtests, the partial effects of the Block design and Information subtests from these multiple linear regression models were report, and their beta values were qualitatively assessed to compare their associations with cognitive performance (second aim).

Four cognitive domains were investigated that have previously been reported in the cohort (Garcia-Cabello et al., 2021), instead of analyzing all the 44 cognitive variables (i.e., 4 models instead of 44 models, per CR proxy) to minimize statistical error type I related to multiple testing. Please see Table 1 for the list of cognitive domains and variables. The procedural memory domain from the cognitive domains in Garcia-Cabello et al. (2021) was excluded for three reasons. Firstly, in the cohort here, the effect of age on procedural memory is weak or non-existing (Correia et al., 2015; Machado et al., 2018; Garcia-Cabello et al., 2021), which is a critical condition for building models related to CR. Secondly, procedural memory is completely disconnected from the other cognitive domains in the cognitive connectome previously built for the cohort (Garcia-Cabello et al., 2021). Since the CR theory relates to compensatory mechanisms with other cognitive networks being recruited to minimize the effect of aging on cognition, this disconnection of procedural memory from the other cognitive domains could pose a barrier in the models of this study. Thirdly, the authors’ focus on the CR and verbal versus non-verbal proxies (presumably left and right brain hemispheres), directs the interest here towards primarily cortical cognitive functions, while procedural memory is more related to the functioning of subcortical brain networks. In addition, since Block design was one of the predictors here, the Block design subtest was excluded from the outcome variable for the visual domain.

Statistical analyses were conducted using the R statistical software (R Core Team, 2016). A value of *p* < 0.05 (two-tailed) was deemed significant in all the analyses.

## 3. Results

**Figure 1:**
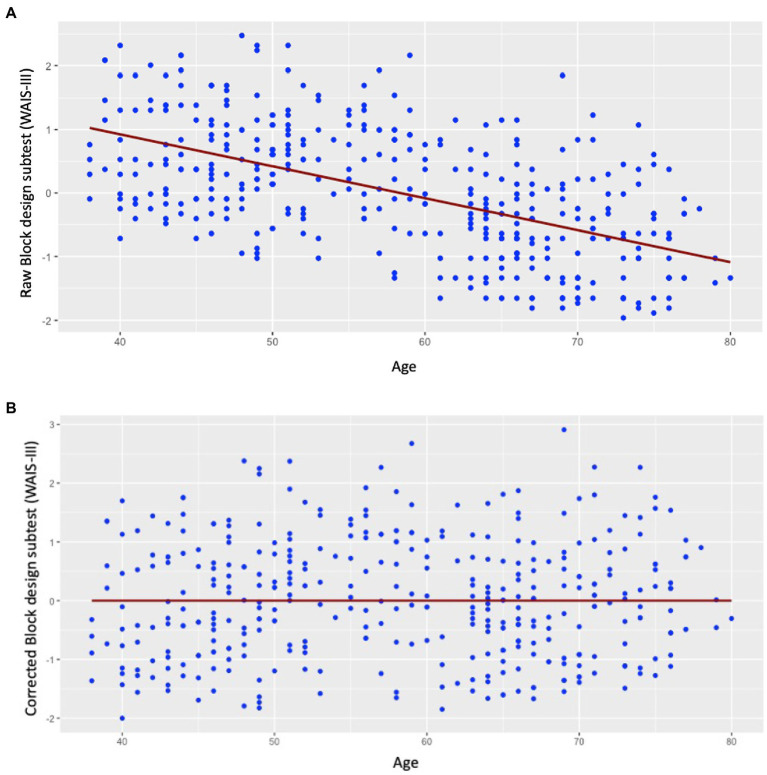
Scatter plots for the correlation of the raw Block design subtest and corrected Block design subtest with age. **(A)** The correlation between the raw Block design and age showed a strong inverse association (*r* = −0.57; *p* < 0.001); **(B)** the correlation between the corrected Block design scores and age is non-significant by design (*r* = 0; *p* = 1.0).

The demographic characteristics of the cohort (*N* = 391) are shown in Table 2. There was a strong inverse association between the raw Block design subtest and age (*r* = −0.572; *p* < 0.001; Figure 1A). After applying the correction from Amato et al. (2006), the new Pearson correlation between the corrected Block design subtest and age demonstrated that the variance of age on Block design was completely removed, as expected (*r* = 0; *p* = 1.0; Figure 1B).

**Table 2:** Demographic characteristics of the sample.

	(A) Whole sample (*n* = 391)		(B) Subsample with data available in the CRQ (*n* = 67)	
	Mean (SD) or percentage	min-max	Mean (SD) or percentage	min-max
Age, years	58.36 (11.34)	38–80	44.64 (3.96)	38–52
Education level (% 0/1/2/3/4)^a^	1/12/37/22/28		0/0/45/34/21	
Sex (% female)	54.40		50.74	
WAIS III-Information	15.26 (6.26)	5–27	15.61 (5.94)	6–26
MMSE	28.51 (1.45)	24–30	29.19 (1.05)	26–30
BDRS	0.89 (1.33)	0–7	0.53 (0.88)	0–3.5

^a^Education Level: illiterate (0); acquired reading and/or writing skills (1); primary level (2); secondary level (3); university level (4). WAIS-III Information: Information subtest of the Wechsler Adult Intelligence Scale. MMSE: Mini-Mental State Examination; BDRS: Blessed Dementia Rating Scale.

### 3.1. Aim 1: Associations between the block design and information subtests, as well as of both proxies with the CRQ

**Figure 2:**
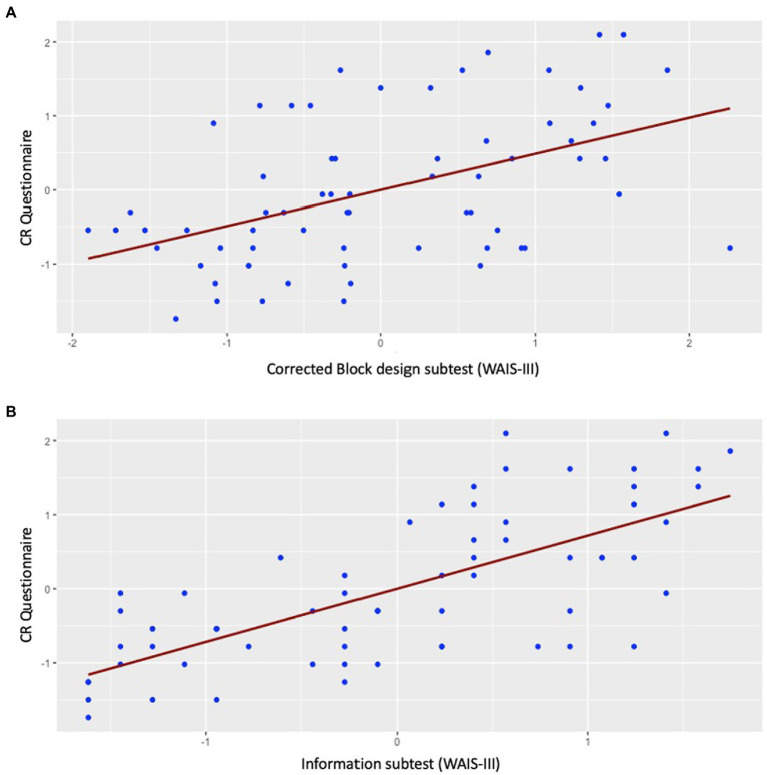
Scatter plots for the correlation of the corrected Block design subtest and the Information subtest with the CR questionnaire. **(A)** The correlation between the corrected Block design and the CR questionnaire showed a positive association (*r* = 0.49; *p* = <0.001); **(B)** the correlation between the Information subtest scores and the CR questionnaire showed a positive association (*r* = 0.72; *p* < 0.001).

The correlation between the corrected Block design subtest and the Information subtest showed a strong direct association (*r* = 0.56; *p* < 0.001). Data on the CRQ was available for 67 participants from a previous study (Ferreira et al., 2016). Table 2B shows the demographic and clinical characteristics of this subsample with data available on the CRQ. In this subsample, the CRQ showed a strong direct correlation with the corrected Block design subtest (*r* = 0.49; *p* = <0.001; Figure 2A) and the Information subtest (*r* = 0.72; *p* < 0.001; Figure 2B).

### 3.2. Aim 2: Associations of the block design and information subtests with cognitive performance

Two series of multiple linear regression models (‘models a’: corrected Block design subtest; ‘models b’: Information subtest) were performed independently to predict each of the four cognitive domains (Table 3). The partial effects of each CR proxy on cognitive performance are described below.

**Table 3:** Multiple linear regression models (models ‘a’ for Block design and models ‘b’ for Information subtest).

Cognitive domains (outcome variables)		*R* ^2^	*F*	*p*	Predictors (*X*)	ß (coef)	*p*	Interaction
								(Pr)
Verbal memory	Model a	0.37	114.70	<0.001	Age	−0.136	<0.001	0.619
					Corrected Block design score	0.091	<0.001	
	Model b	0.44	153.31	<0.001	Age	−0.114	<0.001	0.985
					Information subtest score	0.202	<0.001	
Visual memory–Visuospatial	Model a	0.68	277.91	<0.001	Age	−0.265	<0.001	0.007
					Corrected Block design score	0.198	<0.001	
	Model b	0.65	2.436.28	<0.001	Age	−0.230	<0.001	0.013
					Information subtest score	0.310	<0.001	
Executive functions–Premotor functions	Model a	0.53	148.34	<0.001	Age	−0.363	<0.001	0.030
					Corrected Block design score	0.322	<0.001	
	Model b	0.61	198.35	<0.001	Age	−0.292	<0.001	0.009
					Information subtest score	0.625	<0.001	
Processing speed	Model a	0.37	80.92	<0.001	Age	2.552	<0.001	0.074
					Corrected Block design score	−0.782	<0.001	
	Model b	0.38	87.72	<0.001	Age	2.475	<0.001	0.061
					Information subtest score	−1.795	<0.001	

Regarding the verbal memory domain, both the corrected Block design and the Information subtests predicted performance in the verbal memory domain significantly and independently of age. A higher score in both subtests was associated with a better performance in verbal memory (corrected Block design subtest: *ß* = 0.091; *p* < 0.001; Information subtest: *ß* = 0.202; *p* < 0.001).

When predicting performance in the visual memory and visuospatial domain, a higher score in the corrected Block design and Information subtests was associated with better performance in the visual memory and visuospatial domain performance significantly and independently of age (corrected Block design subtest: *ß* = 0.198; *p* < 0.001; Information subtest: *ß* = 0.310; *p* < 0.001).

Regarding the executive and premotor functions domain, higher scores in the corrected Block design and Information subtests were associated with higher performance significantly and independently of age (corrected Block design subtest: *ß* = 0.322; *p* < 0.001; Information subtest: *ß* = 0.625; *p* < 0.001).

Finally, in the case of the processing speed domain, significant partial effects were found for the corrected Block design and the Information subtest, suggesting that higher scores in both subtests were associated with better processing speed (corrected Block design subtest: *ß* = –0.782; *p* < 0.001; Information subtest: *ß* = –1,795; *p* < 0.001)

The qualitative comparison of the beta values of the corrected Block design and Information subtests showed that, overall, the associations were similar for both proxies, though the beta values were higher for the Information subtest (Table 3).

### 3.3. Aim 3: The capacity of block design to minimize the negative effect of an older age on cognitive performance

**Figure 3:**
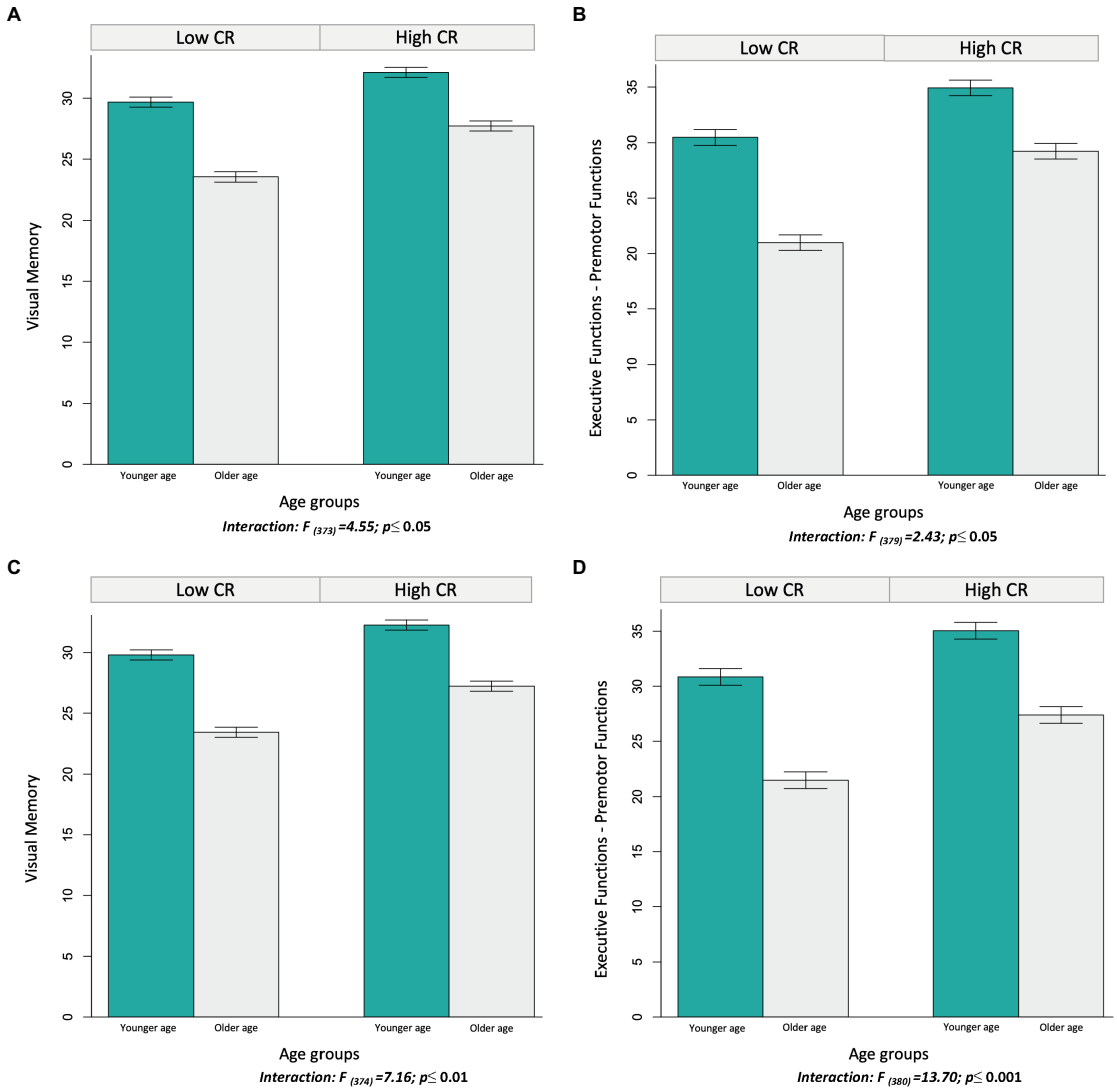
Interactions of the Information subtest and the corrected Block design subtest with age in the prediction of cognitive performance (visual memory and visuospatial domain, and executive functions and premotor domain). All the variables are continuous in the models, but they were dichotomized using the median value for visualization purposes. Bars represent the mean of performance in the different cognitive domains, and the jack-knifes represent 95% confidence intervals. Panels **A** and **B** represent the interaction between the corrected Block design subtest score (median = 33.21) and age (median = 58.36) on the prediction of cognitive domains. Panels **C** and **D** represent the interaction between the Information subtest score (median = 15.27) and age (median = 58.36) on the prediction of cognitive domains.

Two out of the four multiple linear regression models including the corrected Block design subtest and age as predictors showed a significant interaction between both predictors: the models for the visual memory-visuospatial domain and the executive-premotor functions domain (‘models a’ in Table 3). Figures 3A,B show that the older age group obtained a lower performance than the younger age group (visual memory-visuospatial: *t*_(380)_ = 15.074; *p* < 0.0001; executive-premotor functions: *t*_(379)_ = 12.212; *p* < 0.0001), but these differences were smaller in the high cognitive reserve group (visual memory-visuospatial: *t*_(380)_ = 11.930; *p* < 0.0001; executive-premotor functions: *t*_(379)_ = 9.944; *p* < 0.0001).

No statistically significant interactions were found between age and the corrected Block design score for the models predicting verbal memory (*F*_(1,387)_ = 111,70; *p* = 0.619) and processing speed (*F*_(1,387)_ = 80,92; *p* = 0.074) domains (Table 3).

Similarly, out of the four multiple linear regression models including the Information subtest score and age as predictors, two models showed a significant interaction between both predictors. These two models were exactly the same as the corrected Block design subtest, including the visual memory-visuospatial domain and the executive functions-premotor functions domain (‘models b’ in Table 3). The significant interaction between the Information subtest score and age showed exactly the same effect in these two domains as the corrected Block design models did (first paragraph in this section). As can be seen in Figures 3C,D, the older age group had a lower performance than the younger age group (visual memory-visuospatial: *t*_(381)_ = 14.449, *p* < 0.0001; executive-premotor functions: *t*_(380)_ = 13.196, *p* < 0.0001), but these differences were smaller in the high cognitive reserve group (visual memory-visuospatial: *t*_(381)_ = 10.255, *p* < 0.0001; executive-premotor functions: *t*_(380)_ = 7.772, *p* < 0.0001). Similarly, in the corrected Block design subtest, no statistically significant interactions were found between age and the Information subtest score for the models predicting verbal memory (*F*_(1,387)_ = 153,31; *p* = 0.985) and processing speed (*F*_(1,387)_ = 87,72; *p* = 0.061) domains (Table 3).

## 4. Discussion

The present study aimed to determine the potential role of the Block design subtest as a possible non-verbal proxy of CR. The results show that the corrected Block design subtest was strongly correlated with a questionnaire of CR and with the Information subtest, a widely used verbal premorbid IQ proxy of CR. In addition, higher scores in the corrected Block design subtest minimized the negative effect of an older age on cognitive performance in exactly the same cognitive domains as the Information subtest, thus behaving as a CR proxy. The associations of the corrected Block design subtest with cognitive domains were also similar to those of the Information subtest, thus further supporting the similarities between the Block design subtest and the verbal CR proxy of the Information subtest.

The significant correlation between the Block design and Information subtests is consistent with previous findings (Ferreira et al., 2016), where the same cohort was used but the analyses included individuals between 38 and 52 years of age. These previous findings showed that the Block design subtest correlated with cognitive reserve proxies such as WAIS-III Vocabulary and Information subtests, years of education, and the CRQ. Also in the same cohort (Correia et al., 2015) reported that participants between 60 and 80 years of age who performed higher on the Information subtest performed better on the Block design subtest. The present study, thus, managed to generalize the findings from these two previous narrow-age-range studies to a wider age range, from 38 to 80 years of age. This is above all a first piece of evidence in favor of considering the Block design subtest as a potential non-verbal proxy of CR.

Another result of the present study is the significant association of the Block design (and Information subtest) with cognitive performance. In particular, the corrected Block design subtests showed a significant association with the verbal memory domain, the visual memory and visuospatial domain, the executive and premotor functions domain and the processing speed domain, in a such a way that higher scores in Block design were associated with a higher cognitive performance. The association between the Block design subtest and the verbal memory domain may be explained by the strong component of the executive functions of both tasks. It is possible that shared processes such as working memory, planning, developing strategies and processing speed apply in both the Block design and the verbal memory domain. In fact, some authors have reported an important association between verbal memory and executive functions (Duff et al., 2005). The association of Block design with the visual memory and visuospatial domain is reasonable because of the involvement of visuoperceptual, visuospatial and visuoconstructive functions in the Block design performance. The association between the corrected Block design subtest and executive and premotor functions could be interpreted by the participation of premotor functions in the integration of motor skills and learned action sequences (Damasio et al., 2011), which may be involved in Block design performance. Finally, the association between Block design and processing speed is not surprising since the Block design subtest is a timed test with a clear processing speed component (Lezak et al., 2012). Previous studies showed that processing speed predicted a substantial part of the variance in the Block design subtest (Bugg et al., 2006; Schubert et al., 2019).

The associations between the Information subtest and cognitive performance are reminiscent of the associations described above for the corrected Block design subtest. Briefly, the results for the verbal memory domain, visual memory-visuospatial domain, the executive-premotor domain and processing speed domain are consistent with previous studies from the same cohort using different age ranges (Correia et al., 2015; Ferreira et al., 2016). Correia et al. (2015) found a significant effect of the Information subtest scores on verbal memory, visual memory, visuospatial, visuoconstructive, executive and premotor and processing speed tasks. Similarly, Ferreira et al. (2016) found a significant correlation of the Information subtest with verbal memory, visuospatial, visuoconstructive and executive tasks. Taken together, the similar associations obtained for the Block design and Information subtests with cognitive performance serve as another piece of evidence in favor of Block design as a potential non-verbal proxy of CR.

It should be mentioned that the significant interactions between the corrected Block design subtest and age on cognitive performance revealed that higher scores in the corrected Block design subtest minimized the negative effect of age on cognition, in a similar way to the results obtained for the Information subtest. These results are consistent with the predictions of CR theory in normal aging. For example, different studies have demonstrated a slower rate of age-related cognitive decline in individuals with a higher CR (Zahodne et al., 2015). The findings from the interaction of the Information subtest with age in the prediction of cognitive domains enabled the authors to characterize the traditional verbal CR proxy of Information and establish it as a reference for comparison with the proposed non-verbal CR proxy of the corrected Block design.

This study has some limitations. The correlation analysis for the corrected Block design and Information subtests with the CRQ was based on a small subsample of sixty-seven participants whose data were available from a previous study (Ferreira et al., 2016). However, for the purpose of that specific analysis, the statistical power was sufficient to capture the statistically significant association of the corrected Block design subtest with a stablished measure of CR (i.e., the CRQ), which was adequate for the aim of the present study. The inclusion of the CRQ variable made it possible to meet the first aim of the study, and it was not used in further analyses, hence not limiting the number of participants for the analyses in the second and third aims. Another limitation is that potential gender-related differences in cognitive performance and cognitive reserve were not explored. However, to minimize this problem gender differences from the design of this study were controlled for (by setting up a cohort balanced in gender). Schooling years is a common proxy of CR. In this study, the corrected Block design subtest was validated against the Information subtest and the CRQ. These are two well-established proxies of CR and, in particular, the Information subtest performs better than schooling years as a CR proxy in the cohort (Correia et al., 2015; Ferreira et al., 2016). Expanding the present validation here to compare the corrected Block design subtest against schooling years in other different cohorts may be an interesting prospect for future studies. Furthermore, the Block design subtest has both fluid and crystallized components. While the premorbid crystallized component of cognition usually remains stable with aging and in pathology, reflecting fundamental features of CR, the fluid component changes with age. Therefore, the formula proposed by Amato et al. (2006) to statistically remove the variance associated with age in Block design was applied, as a reasonable attempt to remove the fluid component. Although the authors are aware that this is an indirect correction method, there are no strategies in previous research to reduce the fluid component of a given test, to the best of the authors’ knowledge. An alternative strategy could be to isolate the crystallized component of a given test by capturing the common variance between the test (Block design in the present study) and crystallized measures such as the Information subtest. However, such a strategy would completely clash with the design of the present study. Another potential limitation is that the authors accepted the assumption that verbal tests are more related to the functioning of the left hemisphere while non-verbal tests are more related to the functioning of the right hemisphere. Although this assumption is widely accepted in the field, it is almost impossible to definitively prove it. A common strategy to increase the chances that verbal abilities are primarily driven by left hemisphere and non-verbal abilities are primarily driven by right hemisphere is to limit the study population to only include right-handed individuals. The evidence behind this strategy is that around 90% of right-handed individuals have verbal abilities mostly implemented in the left hemisphere (Papadatou-Pastou, 2011). Hence, only right-handed individuals were included in the present study. Furthermore, the study was approached in terms of verbal versus non-verbal proxies, trying to avoid references to brain hemispheric specialization. This is still a valid and important approach because a non-verbal proxy of CR provides the opportunity to estimate CR in populations with low educational levels or with compromised language functions (irrespective of hemispheric specialization). Finally, the Block design subtest was tested as a potential proxy of CR in cognitively unimpaired people. This was necessary to establish the findings in the normal population. Since CR was assessed in the context of increasing age, future studies should expand the present study to demonstrate the potential of Block design as a proxy of CR in individuals with a brain pathology.

In conclusion, it has been demonstrated here that the corrected Block design subtest is significantly associated with two established proxies of CR, it correlates with cognitive performance and high scores in Block design have the capacity to minimize the negative effect of an older age on cognitive performance. Therefore, the results of this study suggest that the corrected Block design subtest could be used as a non-verbal proxy of CR. This could have several clinical implications such as the possibility to estimate CR in patients with verbal impairments or low levels of education.

## Data availability statement

The raw data supporting the conclusions of this article will be made available by the authors, without undue reservation.

## Ethics statement

The studies involving human participants were reviewed and approved by CEIBA; local ethics committee of the University of La Laguna (Spain). The patients/participants provided their written informed consent to participate in this study.

## Author contributions

GC–B contributed to organize the database, performed the statistical analyses, and wrote the first draft of the manuscript. RY-P contributed to the data collection and wrote the first draft of the manuscript. YM contributed to the data collection and to the design of the study, supervised the statistical analyses, participated in the interpretation of the results, supervised the writing of the manuscript, wrote sections of the manuscript, and revised the final version of the manuscript. NC contributed to the data collection, supervised the statistical analysis, and revised the final version of the manuscript. JB contributed to the conception and design of the study, obtained funding, co-supervised the study, and revised the final version of the manuscript. DF contributed to the conception and design of the study, wrote sections of the manuscript, contributed to the interpretation of the results, obtained funding, and supervised the study. All authors have read and approved the submitted version of the manuscript.

## Funding

This research was funded by the Estrategia de Especialización Inteligente de Canarias RIS3 de la Consejería de Economía, Industria, Comercio y Conocimiento del Gobierno de Canarias, co-funded by the Programa Operativo FEDER Canarias 2014–2020 (ProID2020010063), and Universidad Fernando Pessoa Canarias. DF receives funding from the Center for Innovative Medicine (CIMED, 20200505), the regional agreement on medical training and clinical research (ALF, FoUI-962240) between Stockholm County Council and Karolinska Institutet, Hjärnfonden (FO20200066), Alzheimerfonden (AF-968032), Demensfonden, Neurofonden, Stiftelsen För Gamla Tjänarinnor, Karolinska Institutet funding for Research, Karolinska Institutet funding for Geriatric Diseases and Gun och Bertil Stohnes Stiftelse.

## Conflict of interest

The authors declare that the research was conducted in the absence of any commercial or financial relationships that could be construed as a potential conflict of interest.

## Publisher’s note

All claims expressed in this article are solely those of the authors and do not necessarily represent those of their affiliated organizations, or those of the publisher, the editors and the reviewers. Any product that may be evaluated in this article, or claim that may be made by its manufacturer, is not guaranteed or endorsed by the publisher.
